# A foreign body in the cephalic vein: A case report

**DOI:** 10.1097/MD.0000000000011144

**Published:** 2018-06-22

**Authors:** Mingzhi Song, Maohua Wei, Ze Song, Liang Li, Jifeng Fan, Mozhen Liu

**Affiliations:** a Department of Orthopaedics, The First Affiliated Hospital of Dalian Medical University, Dalian; b Department of Orthopaedics, The Third Affiliated Hospital of Dalian Medical University, Jinpu New Area; c Department of General Surgery, The First Affiliated Hospital of Dalian Medical University, Dalian; d Department of General Surgery, The Third Affiliated Hospital of Dalian Medical University, Jinpu New Area, Liaoning, China.

**Keywords:** cephalic vein, foreign body, microsuture, surgery

## Abstract

**Rationale:**

Foreign bodies in the vasculature usually cause numerous problems for clinical physicians. Physician experience with diagnosing and treating non-iatrogenic foreign body migration in the venous system is insufficient.

**Patient concerns:**

Here, we reported a 41-year-old male who had a foreign body in his left forearm following a work-related injury.

**Diagnoses:**

X-ray films indicated a 3-mm high-density shadow in the superficial soft tissue of the left forearm. During the operation, the foreign body was imaged by a C-arm fluoroscope to provide a more accurate location.

**Interventions:**

The foreign body was removed completely following a microsuture of the cephalic vein.

**Outcomes:**

The procedure was uneventful, and the patient remained asymptomatic after 6 months of clinical follow-up.

**Lessons:**

This case indicated that the foreign body in the superficial tissue needed to be accurately diagnosed and located. X-ray and C-arm fluoroscope imaging should be combined with the patient's medical history to ensure sufficient preoperative preparation.

## Abstract 2

Supplemental Digital Content is available in the text

## 1 Introduction

A foreign body (FB) in the vasculature usually causes numerous concerns for clinical physicians, including vascular lesions, infection, bleeding, arrhythmia, and embolism. While most of these consequences are generally caused by migration, FBs in the veins are different from those in the arteries. Due to the direction of blood flow, intravenous FBs can more easily cause lethal pulmonary embolisms, while arterial FBs can cause limb necrosis.^[1,2]^ The former has a low incidence and is often associated with iatrogenic factors such as catheters, cannulas, and needles.^[3–5]^ Non-iatrogenic intravenous FBs, including shrapnel and metallic fragments, are relatively rare emergencies.^[6,7]^ To prevent adverse consequences, rapid tests, accurate diagnosis, and effective extraction of the FB via an operation are particularly important. This uncommon condition can be worrisome for surgeons. Here, aiming to lay a foundation for future clinical work, we comprehensively report one rare case of a non-iatrogenic intravenous FB and a complete review of the related literature.

## 2 Case report

This study was approved by the Ethical Committee of the First Affiliated Hospital of Dalian Medical University. Written informed consent was obtained from the patient.

**Figure 1:**
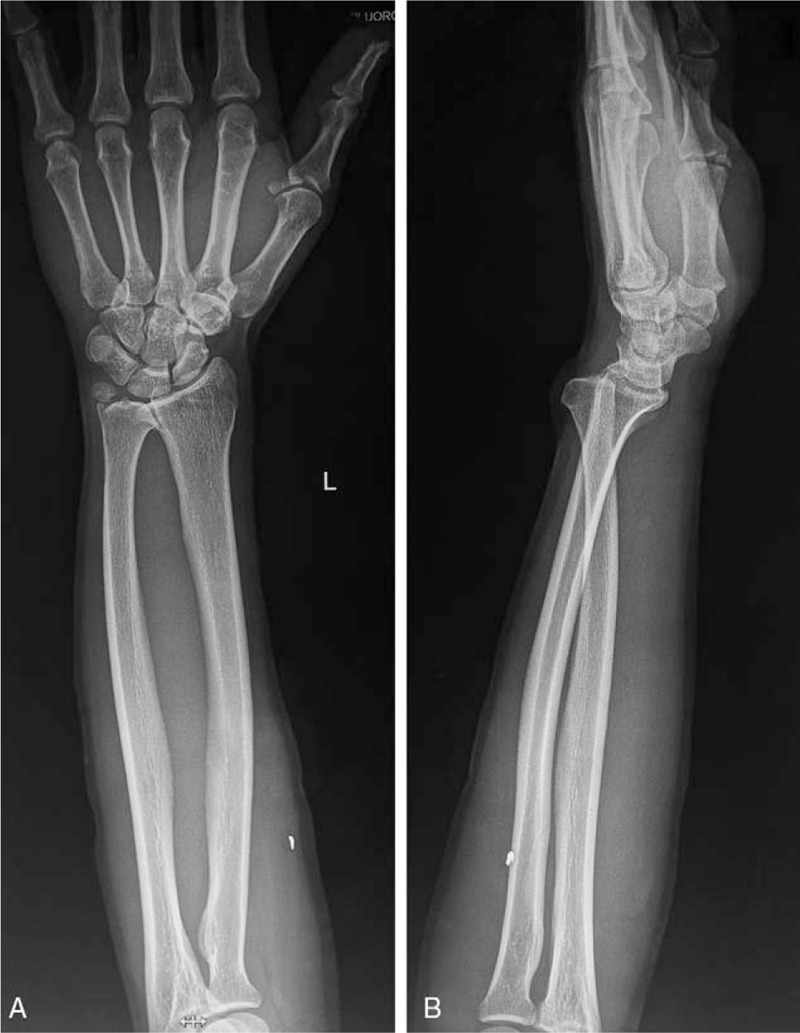
X-ray films of the foreign body. There was a 3-mm high-density shadow in the superficial soft tissue. Additionally, an old fracture of the left ulnar styloid process was found.

A 41-year-old male was admitted to the emergency department with a puncture wound on the palm-side of his left forearm on September 14, 2016. The patient brought 2 X-ray films that indicated that there was a 3-mm high-density shadow in the superficial soft tissue and an existing fracture of the left ulnar styloid process (Fig. 1). His narrative revealed he was injured accidentally by a shard that was flung by a hammer striking a metal nail. The patient immediately applied pressure to the bleeding wound and immediately went to a nearby hospital. Thirty minutes later, the patient came to our center for better treatment.

Clinically, the patient had no signs of a peripheral nerve lesion or hemodynamic disorder. There was no appreciable swelling or hematoma above the forearm. No apparent bleeding could be found around the wound. The patient denied having any other physical discomfort but required surgery to remove the FB. Preoperative examinations were conducted and only the electrocardiographic report produced an unexpected result (sinus bradycardia). The patient had no previous medical history except for an injury in the left wrist injury from an accidental fall.

**Figure 2:**
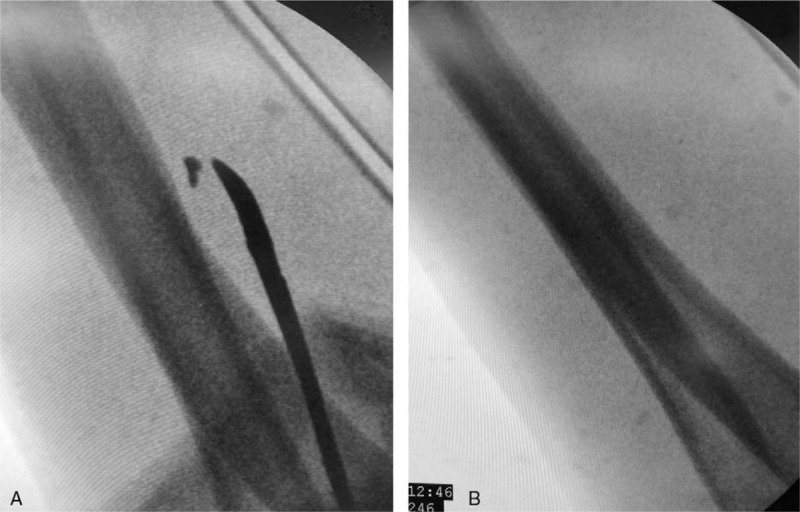
C-arm fluoroscope imaging during the operation. (A) At the beginning of the operation, the location of the foreign body was close to the elbow joint. (B) At the end of the operation, there was no obvious residue remaining.

**Figure 3:**
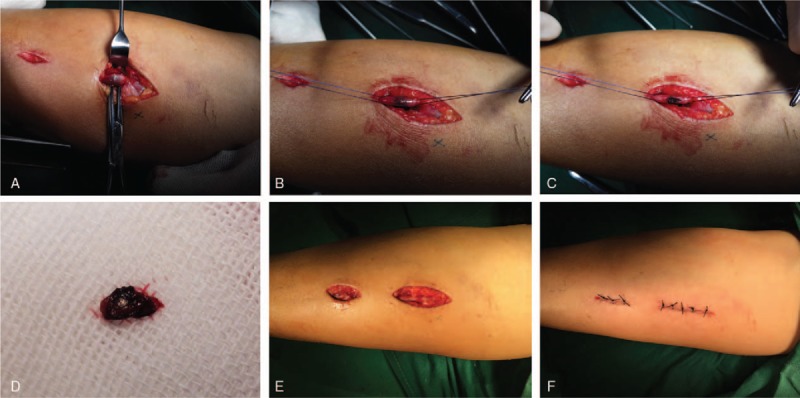
The operation procedure. (A–C) The foreign body (FB) was found and removed from the cephalic vein. (D) The FB was completely displayed. (E,F) The incision of the cephalic vein was repaired by microsuture.

After preoperative preparation, the patient was placed under general anesthesia. A sterile tourniquet was placed for surgical treatment. Extending the left upper limb, the original wound was approximately 5 mm and was enlarged using a No 11 blade. The surgeons then probed the wound and traced the FB layer by layer. Although there was some hematoma surrounding a thicker cephalic vein in the superficial fascia, the deep fascia was still intact. The FB was then imaged using a C-arm fluoroscope, which indicated that the approximate location was closer to the elbow joint than the original wound (Fig. 2A). Based on these results, a new 3-cm incision was made. The superficial tissues were gradually separated by the surgeons until an abnormal vein attracted attention. Though the vessel wall was intact, an unexpected ectogenic substance that seemed not to belong to a vessel was found within the vein. After re-examination with the C-arm fluoroscope, we confirmed that this lesion was the FB. Two sutures were placed to limit the movement of the FB, and a microincision was gently made along the long axis of the vessel (Fig. 3A–C). The FB was removed completely following the trimming of the intima using microscissors and microsutures on venous opening with a 10-0 suture (Ethicon, Inc., Johnson & Johnson, San Lorenzo, Puerto Rico) (Fig. 3D–F). C-arm fluoroscopy was used to examine the region for remnants of the FB, which were not found (Fig. 2B). Therefore, the incisions were closed after washing. The full process lasted 1 hour, and the FB was 3 × 3 × 1 mm in size.

The patient was in good condition after the operation and did not have any cardiopulmonary dysfunction. Radiographic examination of the chest and left forearm on the following day showed no abnormalities. The patient stayed in the hospital for another 5 days and was then discharged. He was recovered well in the 6-month follow-up period.

## 3 Discussion

The FBs in the human body are a common surgical emergency, with an acute onset and rapid progression, usually with severe and complicated clinical symptoms. Commonly, the etiology of these injuries is traumatic or iatrogenic. Trauma usually triggers a complex pathogenesis that can easily result in misdiagnosis or no diagnosis. Gschwind reported a similar case where the FB was initially considered to be lodged in the soft tissues.^[7]^ In fact, the FB had migrated to one of the larger subcutaneous veins. In general, FBs are difficult to find in the narrow passages of organs such as the esophagus, pharynx, bronchus vasculature, and heart. Most intractable types of FBs are those that appear in the vasculature, especially in the venous system. These cases may lead to embolization, infection, cardiac damage, or even death.^[8–11]^ The common types of these FB are needles,^[5]^ intravenous cannulas,^[4]^ guidewires,^[12]^ stents,^[13]^ inferior vena cava filter struts,^[14]^ and other objects (see Table 1).^[6,7,15,16,17–19]^ However, being a rare pathogenic factor, the more unconventional non-iatrogenic FB often requires more extensive treatment and diagnosis.

**Table 1:**
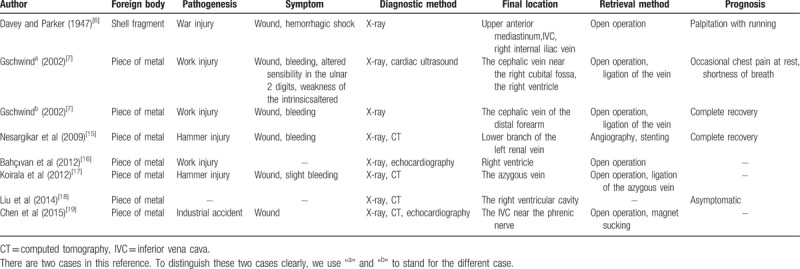
Cases of traumatic intravenous foreign body.

In terms of symptomatology, patients suffering from different intravascular FBs present various symptoms, according to the position of the FB. However, patients are usually asymptomatic,^[10,18]^ as was seen in the patient in this case report. Among symptomatic patients with FBs, local pain and discomfort are the most common clinical manifestations. Shock arising from losing a large amount of blood may also occur in combat trauma patients.^[6]^

The diagnostic method for an FB in a vein is relatively simple. X-ray photography, computed tomography, and angiography are commonly performed. Nevertheless, the diagnostic validity of these methods depends on the properties and density of the FB. There are 2 main types of FB: high-density FBs and low-density FBs. Metal fragments can be found by more-sensitive imagologic examinations.^[7,8,19]^ Contrastingly, low-density FBs rely more on angiography. If none of the above-mentioned methods are successful, directly retrieving the FB by operation or vascular intervention is the last choice.

There are 3 main aims of treatment: saving lives, removing the FB(s), and reducing relative complications. Therapeutic types fall into 2 categories: invasive treatments (such as operations and vascular interventions) and conservative treatments (based on patient requirements). Combining the previous report with our experience, we created an animation that depicts the pathogenesis of this rare FB (see Video 1). In general, open operations have commonly been performed to remove FBs lodged in superficial veins.^[7,19]^ For vascular interventions, minimally invasive surgical technology seems more proficient for interventional radiologists. This method of retrieval is particularly useful for FBs in the heart.^[8]^ In a previous report, surgeons were inclined to ligate the damaged veins.^[6,7]^ However, in this case, we used a microscopic suture technique to repair the cephalic vein and minimized the damage to the patient. Additionally, anti-infection treatment and anticoagulation are both equally important to ensure the success of the therapy.^[7,8]^ Follow-up can evaluate the patient's status and detect the outcome of the treatment.^[18]^

## 4 Conclusion

Intravenous FB migration in the venous system is rare. These cases are commonly triggered by traumatic and iatrogenic factors. For traumatic cases, both the cause of the injury and the radiographic results should be considered to make a correct diagnosis. More attention should be given to monitoring the vital signs of patients. The key preconditions for treatment are respecting the patient's attitude, removing the FB and protecting the function of the affected area. Postoperative anticoagulation and anti-infection therapy are essential. The patient should receive follow-up care to observe their prognosis. This case report indicated that an FB in the superficial tissue required diagnosis and accurate location. X-ray and C-arm fluoroscope imaging should be combined with the patient's medical history to ensure that sufficient preoperative preparations are made.

## Author contributions

MS contributed to diagnosing the case, performing the operation, seeking references and manuscript writing. JF and ML were responsible for our manuscript. MW contributed to the critical revision of the manuscript for intellectual content. ZS and LL helped to write and to revise the manuscript. All authors read and approved the final manuscript.

**Conceptualization:** Mingzhi Song.

**Methodology:** Mingzhi Song, Mozhen Liu.

**Supervision:** Jifeng Fan, Mozhen Liu.

**Writing – original draft:** Mingzhi Song.

**Writing – review & editing:** Mingzhi Song, Maohua Wei, Ze Song, Liang Li.

## Supplementary Material

Supplemental Digital Content
